# Histone deacetylase 8 protects human proximal tubular epithelial cells from hypoxia-mimetic cobalt- and hypoxia/reoxygenation-induced mitochondrial fission and cytotoxicity

**DOI:** 10.1038/s41598-018-29463-x

**Published:** 2018-07-27

**Authors:** Soon-Duck Ha, Ori Solomon, Masoud Akbari, Alp Sener, Sung Ouk Kim

**Affiliations:** 1 Department of Microbiology & Immunology and Infectious Diseases Research Group, Siebens-Drake Research Institute, Schulich School of Medicine & Dentistry, University of Western Ontario, 1400 Western Road, London, Ontario N6G 2V4 Canada; 2 Department of Surgery, Schulich School of Medicine & Dentistry, University of Western Ontario, London, Ontario N6G 2V4 Canada

## Abstract

Cell death by hypoxia followed by reoxygenation (H/R) is responsible for tissue injury in multiple pathological conditions. Recent studies found that epigenetic reprogramming mediated by histone deacetylases (HDACs) is implicated in H/R-induced cell death. However, among 18 different isoforms comprising 4 classes (I-IV), the role of each HDAC in cell death is largely unknown. This study examined the role of HDAC8, which is the most distinct isoform of class I, in the hypoxia mimetic cobalt- and H/R-induced cytotoxicity of human proximal tubular HK-2 cells. Using the HDAC8-specific activator TM-2-51 (TM) and inhibitor PCI34051, we found that HDAC8 played a protective role in cytotoxicity. TM or overexpression of wild-type HDAC8, but not a deacetylase-defective HDAC8 mutant, prevented mitochondrial fission, loss of mitochondrial transmembrane potential and release of cytochrome C into the cytoplasm. TM suppressed expression of dynamin-related protein 1 (DRP1) which is a key factor required for mitochondrial fission. Suppression of DRP1 by HDAC8 was likely mediated by decreasing the level of acetylated histone H3 lysine 27 (a hallmark of active promoters) at the DRP1 promoter. Collectively, this study shows that HDAC8 inhibits cytotoxicity induced by cobalt and H/R, in part, through suppressing DRP1 expression and mitochondrial fission.

## Introduction

Hypoxia followed by reoxygenation (H/R) is an event characterized by the restriction and subsequent restoration of blood flow to an organ. H/R is the main cause of extensive tissue damage that ensues in multiple clinical scenarios, such as myocardial infarction, ischemic stroke, trauma, sickle cell diseases, sleep apnea, sepsis, solid organ transplantation and major surgery^1^. In the kidney, H/R is implicated in renal tubular cell death which can later manifest as acute kidney injury and end-stage renal disease^2^. To date, much progress has been made in understanding the cellular and molecular mechanisms of H/R-induced tissue damage. However, effective agents for preventing or treating such events are yet to be developed.

One of the main outcomes of H/R is activation of cell death pathways resulting from alterations in gene expression. Particularly, gene transcription regulated by epigenetic reprogramming mediated through modifying acetylation at the N-terminus of histones has been shown to be involved in the pathogenesis of acute kidney injury^3,4^. The level of histone acetylation is determined by two counteracting enzymes: histone acetyltransferases and histone deacetylases (HDACs). In mammals, 18 isoforms of HDACs have been identified with four different classes based on their sequence homology to yeast HDACs: class I (HDAC1, 2, 3 & 8), class II (HDAC 4–7, 9 & 10), class III (SIRT1-7) and class IV (HDAC11). Among them, class I HDACs, which are localized in the cell nucleus, remove acetyl groups from ε-N-acetyl-lysine of histones and interact with co-repressors that lead to chromatin condensation and gene repression^5^. Within class I HDACs, HDAC8 is the most divergent isoform with distinct subcellular localization, substrate recognition, post-translational modifications and sensitivity to class I inhibitors^6^.

Several recent studies have demonstrated that HDACs are involved in ischemia-reperfusion injury of the brain and heart, so targeting HDACs, particularly class I HDACs, has been suggested to be a potential therapeutic strategy^7–9^. Although contradictory results have been reported^10,11^ for the kidney, broad and class I-specific HDAC inhibitors were shown to be beneficial for cell survival and recovery from tissue damage during acute kidney injury^3,12,13^. However, these studies used pan-specific inhibitors, such as suberoylanilide hydroxamic (SAHA) and trichostatin, or the class I inhibitor MS-275 that has no effect on HDAC8^14^. Therefore, the role of HDAC8 in kidney cell death remains unknown.

This study examined the role of HDAC8 in H/R-induced kidney cell viability using human renal proximal tubular HK-2 cells. Here, we showed that the HDAC8-specific activator TM^15^ or ectopic expression of wild-type HDAC8, but not a catalytically defective HDAC8 mutant, prevented mitochondrial fission and dysfunction induced by cobalt^16–18^ and H/R. These results suggest that HDAC8 plays a protective role in H/R-induced cytotoxicity in kidney tubular epithelial cells.

## Results

### HDAC8 protects HK-2 cells from cytotoxicity induced by cobalt and H/R

**Figure 1:**
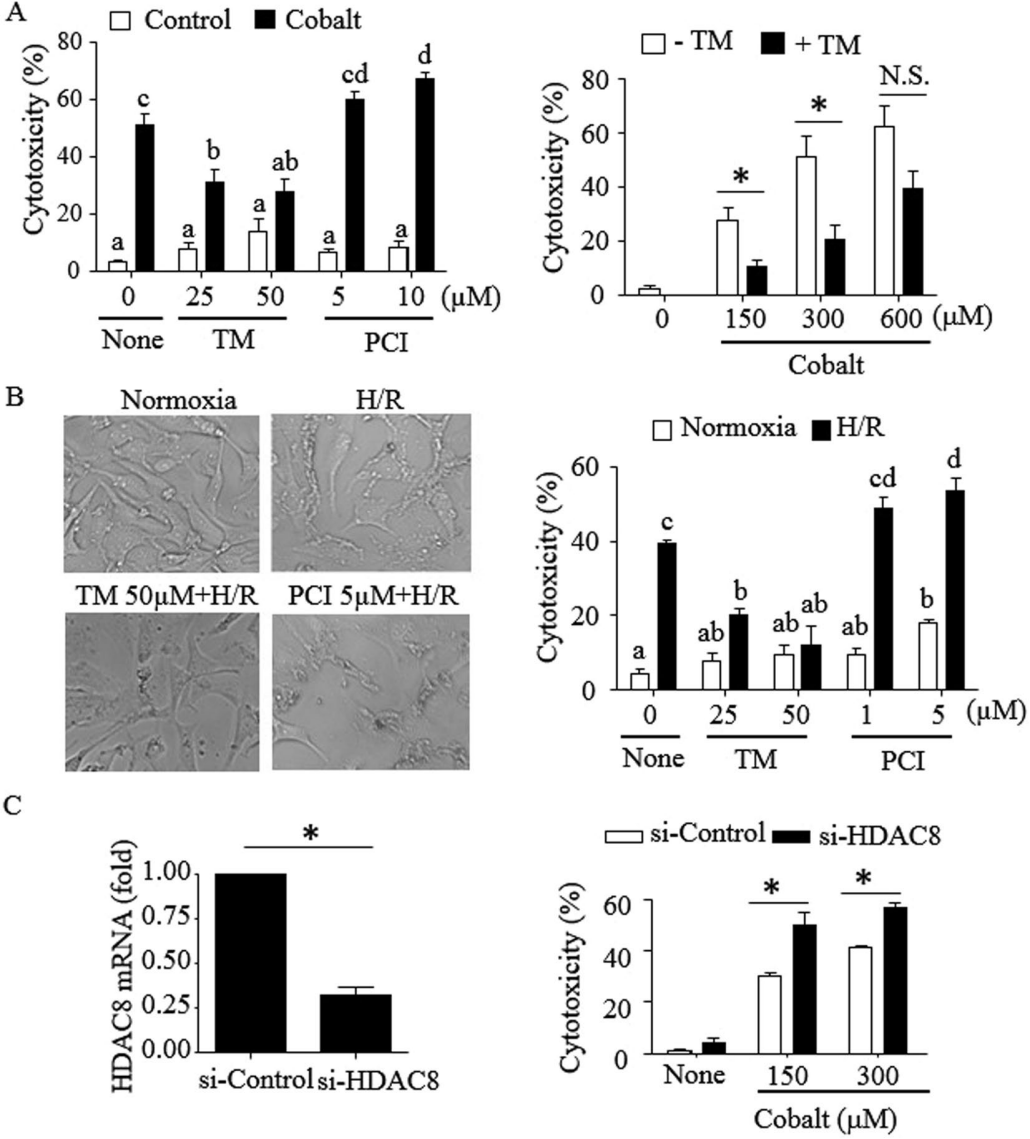
The HDAC8 activator TM-2-51 protects HK-2 cells from cytotoxicity induced by cobalt and hypoxia/reoxygenation (H/R). (**A**) HK-2 cells were treated with cobalt (CoCl_2_; 300 µM) in the presence or absence of TM-2-51 (TM), the HDAC8 inhibitor PCI-34051 (PCI) and drug vehicle DMSO for 20–22 h (left panel). Similarly, cells were treated with varying doses of cobalt in the presence or absence of 25 µM TM (right panel). (**B**) Cells were incubated in the hypoxia chamber (0.2% O_2_, 5% CO_2_ & 95% N_2_ at 37 °C) for 24 h, followed by a standard cell culture incubator (~21% O_2_, 5% CO_2_ & ~74% N_2_ at 37 °C) for the next 16–18 h. Cells were treated with or without TM (25 & 50 µM) or PCI (1 & 5 µM) throughout the experimental period. Cells were observed by microscopy using an Olympus microscope and images were acquired using QCapture Pro software at 20x magnification (left panel). (**C**) HK-2 cells transfected with control (scrambled) siRNA or HDAC8-specific siRNAs for 24 h were exposed to cobalt (300 µM) for 16–20 h. HDAC8 siRNA was validated by real-time PCR analysis at 48 h post-transfection (left panel). (**A**–**C**) Cytotoxicity was measured by MTT assay as described in “*Methods*”. Results are from 3 independent experiments and data are expressed as mean ± S.D. For statistical analysis, Student’s *t* tests were performed on the data presented in A right and C panels (*p < 0.05; N.S., not significant), and one-way ANOVA with Tukey multiple comparison tests were performed on data presented in A left and B right panels (p < 0.05; columns accompanied by the same letter are not significantly different from each other).

To examine the role of HDAC8 in H/R-induced cytotoxicity, human renal proximal tubular HK-2 cells were treated with cobalt in the presence or absence of the HDAC8 activator TM and inhibitor PCI-34051 (PCI)^19^, and cell viability was measured using an MTT assay. Cobalt (300 µM) induced ~50% cytotoxicity in 20–22 h (Fig. 1A, left panel). TM significantly prevented the cytotoxic effect of cobalt up to 30–40% at 25–50 µM concentrations; whereas, PCI slightly but significantly enhanced cytotoxicity at 10 µM concentration. The protective effect of TM was observed in a range of cobalt concentrations up to 300 µM (Fig. 1A, right panel). At 600 µM of cobalt, the protective effect of TM did not reach statistical significance. To further examine the role of HDAC8 in H/R-induced cytotoxicity, HK-2 cells were cultured in a hypoxic environment (0.2% O_2_) for 24 h with subsequent reoxygenation at atmospheric O_2_ (~21%) for 16–18 h. Under these hypoxic conditions, loss of cell integrity became apparent which was more pronounced in the presence of PCI compared to TM (Fig. 1B, left panel). Consistent with these qualitative observations, H/R induced ~40% cytotoxicity, which was significantly increased to ~55% cytotoxicity and decreased to ~15% cytotoxicity in the presence of PCI (5 µM) and TM (50 µM), respectively (Fig. 1B, right panel). To further confirm the protective role of HDAC8, sensitivity to cobalt- and H/R-induced cytotoxicity was measured after knocking down HDAC8 by small interfering (si) RNA. The siRNA knocked down ~75% of HDAC8 mRNAs (Fig. 1C, left panel). Consistent with the cytotoxic effect of PCI, cells treated with the HDAC8-targeting siRNA were more susceptible to cytotoxicity induced by cobalt than cells treated with scrambled siRNA (Fig. 1C, right panel). Similar protective and promoting effects of TM and PCI, respectively, on cytotoxicity induced by cobalt were also observed in rat renal proximal tubular NRK-52E cells (Supplemental Fig. S1), suggesting that the protective role of HDAC8 was not limited to HK-2 cells.

### HDAC8 protects HK-2 cells from mitochondrial dysfunction induced by cobalt and H/R

**Figure 2:**
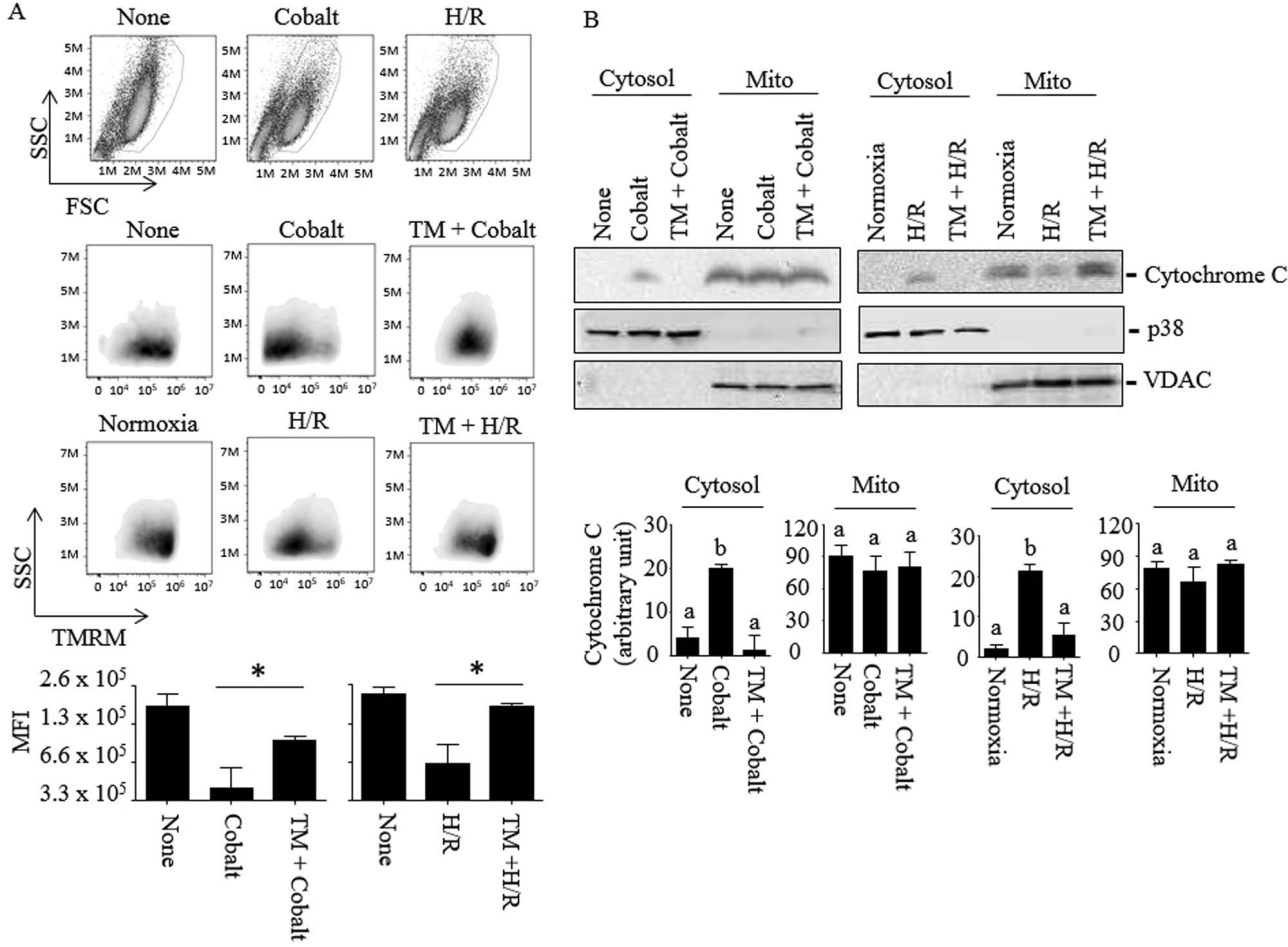
TM protects HK-2 cells from mitochondrial membrane dysfunction induced by cobalt and H/R. (**A**) HK-2 cells were exposed to nothing, cobalt (300 µM) for 16–18 h or H/R [incubated in the hypoxia chamber (0.2% O_2_, 5% CO_2_ & 95% N_2_ at 37 °C) for 24 h, then in a standard cell culture incubator (~21% O_2_, 5% CO_2_ & ~74% N_2_ at 37 °C) for the next 6 h] in the presence or absence of TM (25 µM). Cells were then stained with the mitochondrial membrane potential probe TMRM and analyzed by FACS as described in “*Methods*”. Cells with normal morphological features, based on forward and side scattered light parameters, were gated for mitochondrial membrane depolarization measurement (top panel, circled). TMRM fluorescence (middle panel) and median fluorescence intensities (bottom panel) were plotted using the FlowJo software. FACS images shown are representative of three (cobalt) and two (H/R) independent experiments. Data are expressed as mean ± S.D. (*p < 0.05; Student’s *t*-test). (**B**) Similarly, HK-2 cells were treated with cobalt or H/R and the cytosolic and mitochondrial fractions prepared as described in “*Methods*”. Release of cytochrome C from mitochondria to the cytosol was measured by immunoblots (upper panel). Immunoblots against anti-p38 and anti-VDAC were used for loading controls and markers for differential cell lysate preparations. Images shown are representative of three independent experiments. Cytochrome C band intensities relative to corresponding loading controls were analyzed by NIH images J program (lower panel). Data are expressed as mean ± S.D. [p < 0.05 by one-way ANOVA with Tukey multiple comparison test; columns accompanied by the same letter are not significantly different from each other].

Mitochondrial dysfunction and release of cytochrome C into the cytosol induced by cobalt^20,21^ and H/R^22^ trigger programmed cell death. Therefore, we examined if HDAC8 inhibited cytotoxicity through preventing mitochondrial dysfunction. HK-2 cells were exposed to cobalt and H/R in the presence or absence of TM, and the mitochondrial transmembrane potential (ΔΨm) was examined by flow cytometry using the mitochondrial fluorescent dye TMRM (tetramethylrhodamine methyl ester). Cells with normal granularity (side scatter light intensity) and size (forward scatter light intensity) were gated (Fig. 2A, top panel) and measured for TMRM intensity (middle panel). The median fluorescence intensity (MFI) of cells exposed to cobalt and H/R were lower than that of non-treated cells (bottom panel). TM significantly enhanced the MFI of TMRM in cells exposed to cobalt and H/R. Similarly, cytochrome C, which was absent in the cytosolic fractions of non-treated cells, was detected in cells exposed to cobalt and H/R (Fig. 2B, top panel). The mitochondrial cytochrome C levels were not significantly different among these cells, suggesting that a portion of cytochrome C is translocated from mitochondria to the cytoplasm by cobalt and H/R. TM significantly prevented the release of cytochrome C into the cytoplasm in cobalt- and H/R-exposed cells (Fig. 2B, bottom panel). Immunoblots for the mitochondrial voltage-dependent anion channel (VDAC) and cytosolic p38 showed minimal cross-contamination between cytosolic and mitochondrial fractions (Fig. 2B, top panel).

### HDAC8 prevents mitochondrial fission induced by Cobalt and H/R

**Figure 3:**
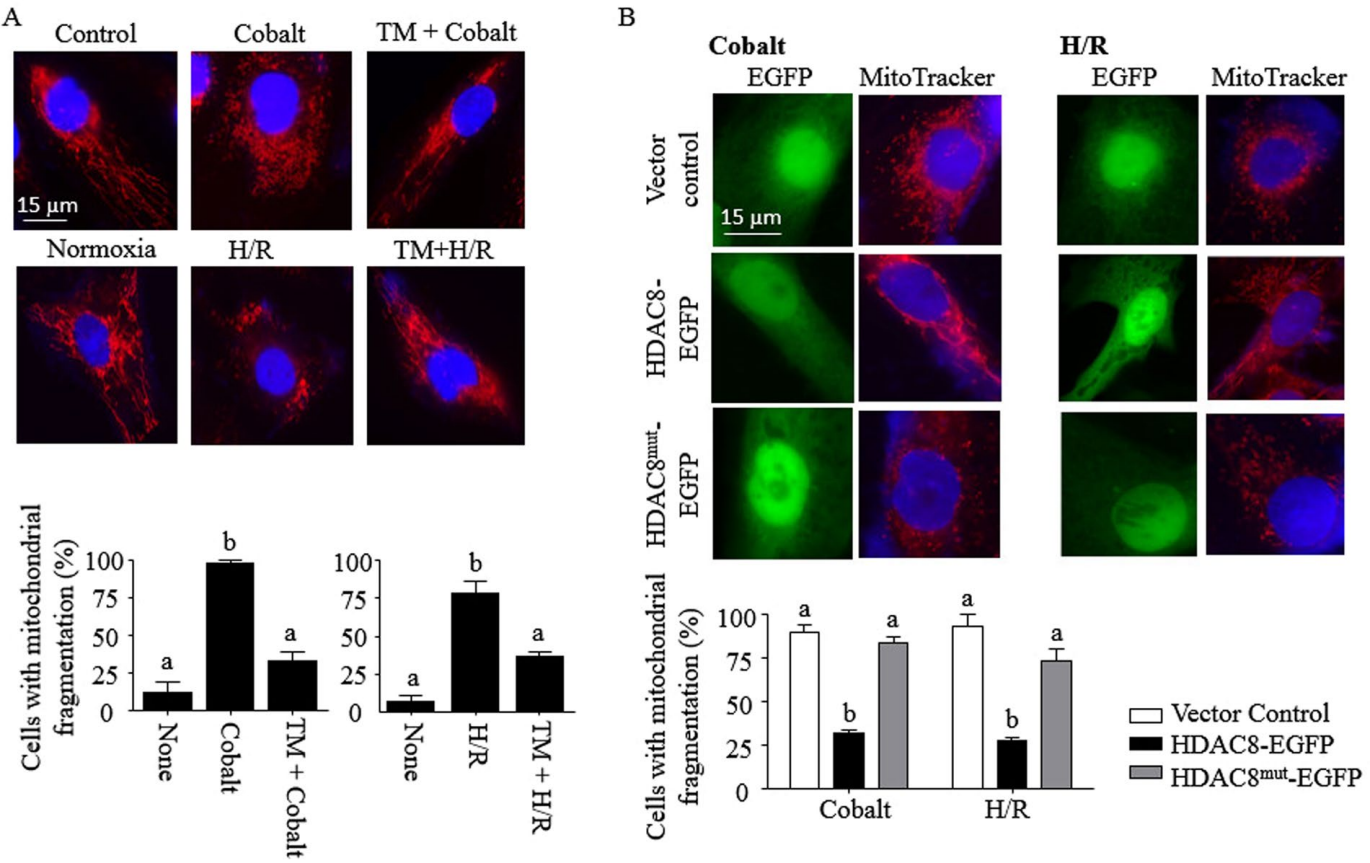
HDAC8 inhibits mitochondrial fragmentation induced by cobalt and H/R in HK-2 cells. (**A**,**B**) HK-2 cells (A) and HK-2 cells transfected with EGFP (vector control), HDAC8 conjugated with EGFP (HDAC8-EGFP) or deacetylase-defective mutant HDAC8 conjugated with EGFP (HDAC8^mut^-EGFP) vectors (B) were exposed to cobalt (300 µM) for 16–18 h or incubated in the hypoxia chamber (0.2% O_2_, 5% CO_2_ & 95% N_2_ at 37 °C) for 24 h, followed by a standard cell culture incubator (~21% O_2_, 5% CO_2_ & ~74% N_2_ at 37 °C) for the next 6 h. Cells were treated with or without TM (25 µM) throughout the experimental period. These cells were then stained with MitoTracker^®^-Red as described in “*Methods*”. Cells were visualized (600x magnification) and representative images of three independent experiments are shown. A total of 50 randomly selected cells (**A**) or 25 EGFP-expressing cells (**B**) from each sample were evaluated for mitochondrial fission. Data are expressed as mean % of cells with apparent mitochondrial fission ± S.D. [n = 3; p < 0.05 by one-way ANOVA with Tukey multiple comparison test; columns accompanied by the same letter are not significantly different from each other].

**Figure 4:**
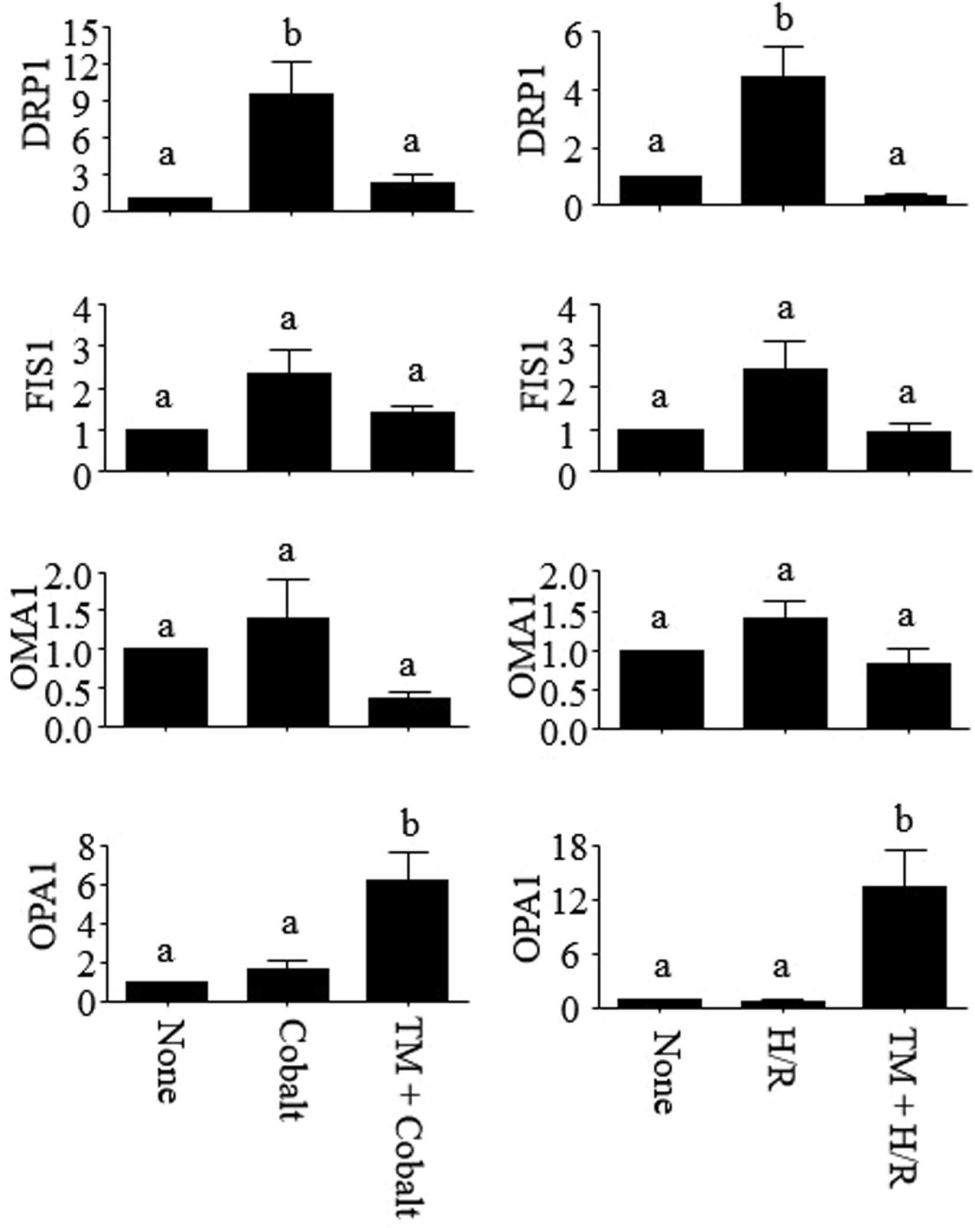
HDAC8 regulates expression of genes involved in mitochondrial fission and fusion. HK-2 cells were exposed to Cobalt (300 µM; 14–16 h) or incubated in the hypoxia chamber (0.2% O_2_, 5% CO_2_ & 95% N_2_ at 37 °C) for 24 h, followed by a standard cell culture incubator (~21% O_2_, 5% CO_2_ & ~74% N_2_ at 37 °C) for the next 5 h (H/R). Expression of DRP1, FIS1, OMA1 and OPA1 mRNAs were analyzed by qPCR. Data are expressed as mean ± S.D. [n = 3; p < 0.05 by one-way ANOVA with Tukey multiple comparison test; columns accompanied by the same letter are not significantly different from each other].

Mitochondrial fission induces or enhances cell death in cells exposed to cobalt and ischemia-reperfusion^23,24^. We also found that both cobalt and H/R induced mitochondrial fragmentation in 90% and 70% of HK-2 cells, respectively, which could be prevented by TM (Fig. 3A). To further confirm a protective role of HDAC8 in mitochondrial fragmentation, cells were transfected with EGFP-conjugated wild-type and deacetylase-defective mutant HDAC8 expression vectors. On average, transfection efficiency in these cells reached ~20%. These EGFP-positive cells were analyzed for mitochondrial fragmentation (Fig. 3B, top panel). Ectopic overexpression of wild-type, but not the mutant, HDAC8 prevented mitochondrial fragmentation induced by cobalt and H/R (Fig. 3B, bottom panel). We then examined whether HDAC8 regulated expression of genes known to be involved in mitochondrial fission (*DRP1* and *FIS1*) and fusion (*OMA1* and *OPA1*)^25^. Indeed, cobalt and H/R induced DRP1 expression, which was completely inhibited by TM (Fig. 4, top panel). Cobalt and H/R alone had no significant effects on the expression of FIS1 (mitochondrial fission 1), OMA1 (zinc metallopeptidase) and OPA1 (mitochondrial dynamin like GTPase). However, in the presence of TM, cobalt and H/R significantly induced OPA1 expression (Fig. 4, bottom panel). Collectively, these results suggest that HDAC8 regulates expression of genes involved in mitochondrial fusion and fission that leads to mitochondrial fusion.

### Inhibition of DRP1 protects HK-2 cells from mitochondrial dysfunction and fission, and cytotoxicity

**Figure 5:**
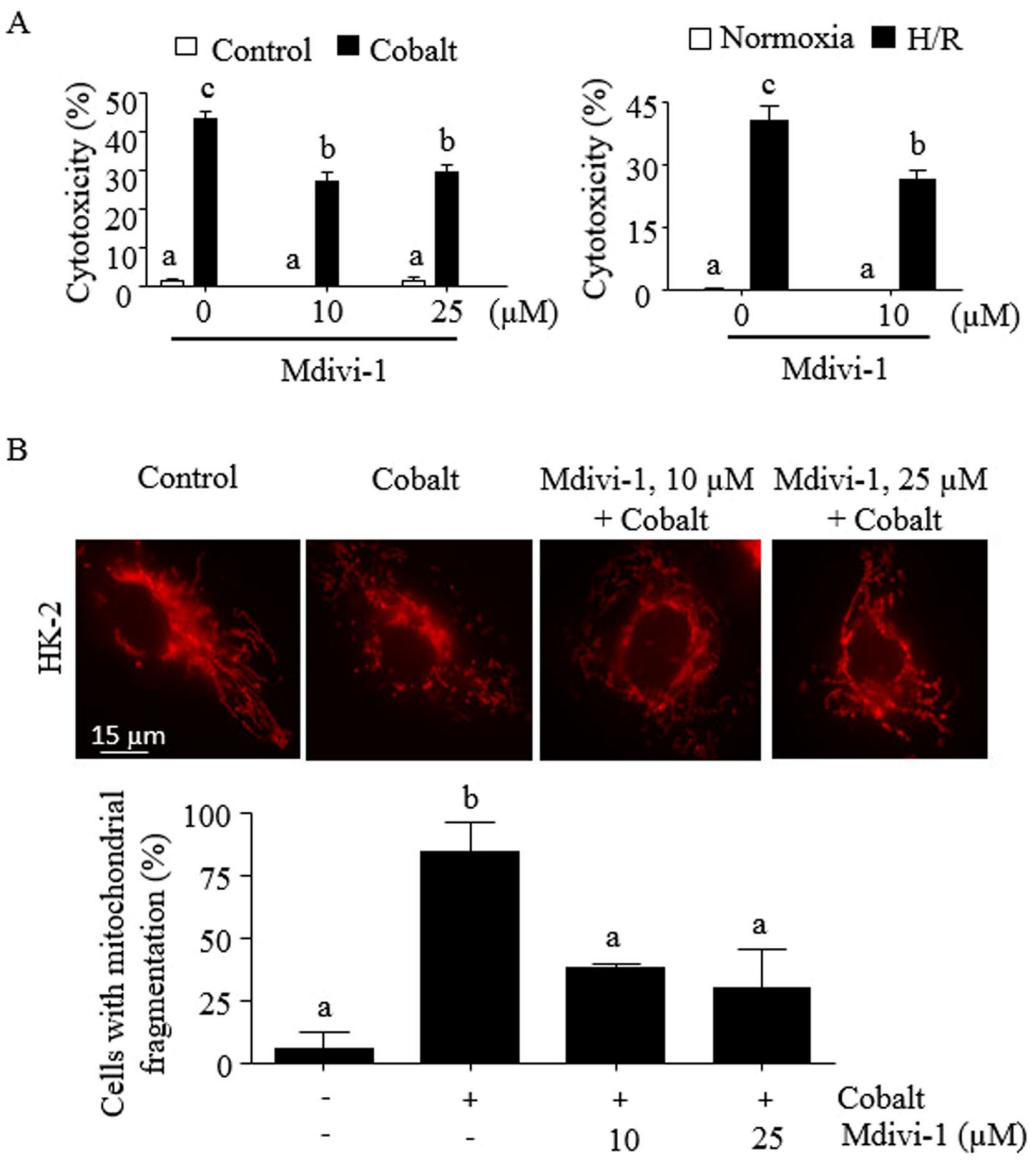
The DRP1 inhibitor Mdivi-1 protects HK-2 cells from mitochondrial fission and cytotoxicity induced by cobalt and H/R. (**A**) HK-2 cells were treated with cobalt (300 µM) for 16 h (left panel) or incubated in the hypoxia chamber (0.2% O_2_, 5% CO_2_ & 95% N_2_ at 37 °C) for 24 h, followed by a standard cell culture incubator (~21% O_2_, 5% CO_2_ & ~74% N_2_ at 37 °C) for the next 16 h in the presence or absence of Mdivi-1. Cytotoxicity was measured by an MTT assay as described in “*Methods*”. (**B**) Similarly, HK-2 cells were treated with cobalt (300 µM) in the presence or absence of Mdivi-1 for 16 h and stained with MitoTracker^®^-Red. Cells were then visualized (600x magnification) and a representative image of 3 independent experiments is shown (top panel). A total of 30 randomly selected cells from each sample was evaluated for mitochondrial fission (bottom panel). (**A**,**B**) Data are expressed as mean ± S.D. [n = 3; p < 0.05 by one-way ANOVA with Tukey multiple comparison test; columns accompanied by the same letter are not significantly different from each other].

**Figure 6:**
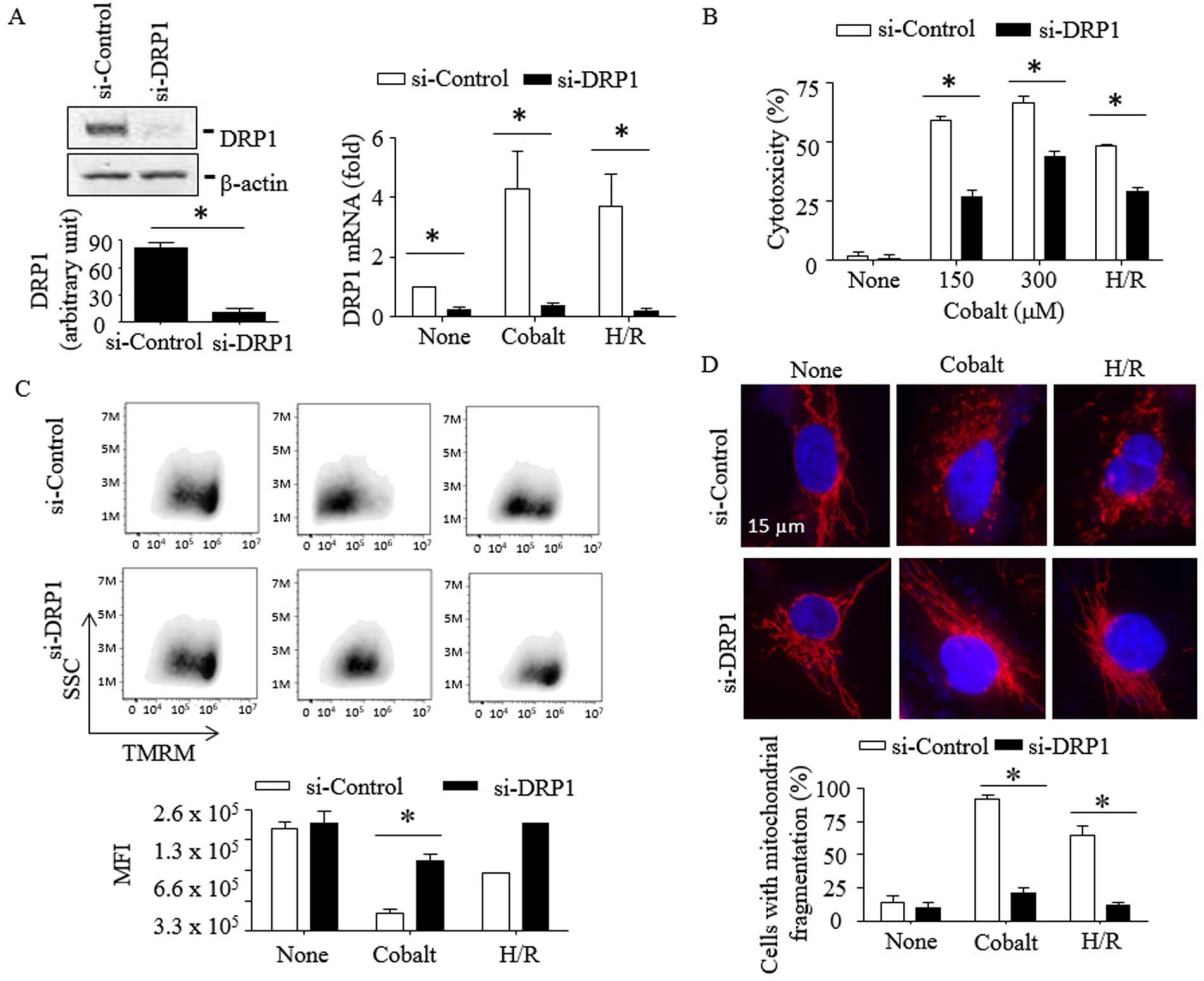
Knocking down DRP1 protects HK-2 cells from mitochondrial fission, mitochondrial transmembrane depolarization and cytotoxicity induced by cobalt and H/R. HK-2 cells were transfected with control or DRP1-specific siRNAs for 24 h as described in “*Methods*”. Cells were then exposed to cobalt (300 µM) for 16–20 h or incubated in the hypoxia chamber (0.2% O_2_, 5% CO_2_ & 95% N_2_ at 37 °C) for 24 h, followed by a standard incubator (~21% O_2_, 5% CO_2_ & ~74% N_2_ at 37 °C) for the next 6 h (**A**,**C**,**D**) or 16–20 h (**B**). (**A**,**B**) Knocking down DRP1 by siRNA was confirmed by Western blots using anti-DRP1 antibody at 48 h after transfection (**A**, left-upper panel). Immunoblots shown are representative of three independent experiments. DRP1 band intensities relative to those of β-actin were analyzed by NIH images J program (A, left-lower panel). DRP1 mRNA expression level (**A**, right panel) and cytotoxicity (**B**) were measured by real-time PCR analysis and MTT assay, respectively. Data are expressed as mean ± S.D. (n = 3; *p < 0.05; Student’s *t*-test). (**C**) Mitochondrial transmembrane potential was measured as described in the “*Methods*”. TMRM median fluorescence intensity (MFI) was calculated using FlowJo. Data shown in cobalt are representative of two independent experiments and H/R is a single experiment. *p < 0.05 (Student’s *t*-test). (**D**) For visualization of mitochondria, cells were then stained with MitoTracker^®^-Red and observed by fluorescence microscopy. Images shown are representative of three independent experiments (top panel). A total of 50 randomly selected cells from each sample was evaluated for mitochondrial fission (bottom panel). Data are expressed as mean ± S.D. (n = 3; p < 0.05; Student’s *t*-test).

Since expression of DRP1 was induced by cobalt and H/R (Fig. 4), the role of DRP1 in mitochondrial fission and cell viability in HK-2 cells was further examined. First, we examined the effect of the DRP1 inhibitor Mdivi-1^26^ on cytotoxicity and mitochondrial fission. Mdivi-1 at 10 µM significantly prevented cytotoxicity (Fig. 5A) and mitochondrial fission (Fig. 5B) in cells exposed to cobalt and H/R. Similarly, Mdivi-1 prevented cytotoxicity and mitochondrial fission in rat renal proximal tubular NRK-52E cells (Supplemental Fig. S2). We then used siRNA to further confirm the role of DRP1 in these cells. The siRNA targeting DRP1 was able to decrease mRNA and protein levels of DRP1 up to ~90% in non-treated, as well as cobalt- and H/R-treated cells (Fig. 6A). Knocking down DRP1 by siRNA also reduced cytotoxicity induced by cobalt and H/R by ~45% (Fig. 6B), which was comparable to that by TM (Fig. 1). Knocking down DRP1 also significantly reduced the loss of ΔΨm (Fig. 6C) and mitochondrial fragmentation (Fig. 6D).

### HDAC8 selectively reduces the level of acetylated histone H3 lysine 27 residue (H3K27ac) associated with the DRP1, but not OPA1, promoter

**Figure 7:**
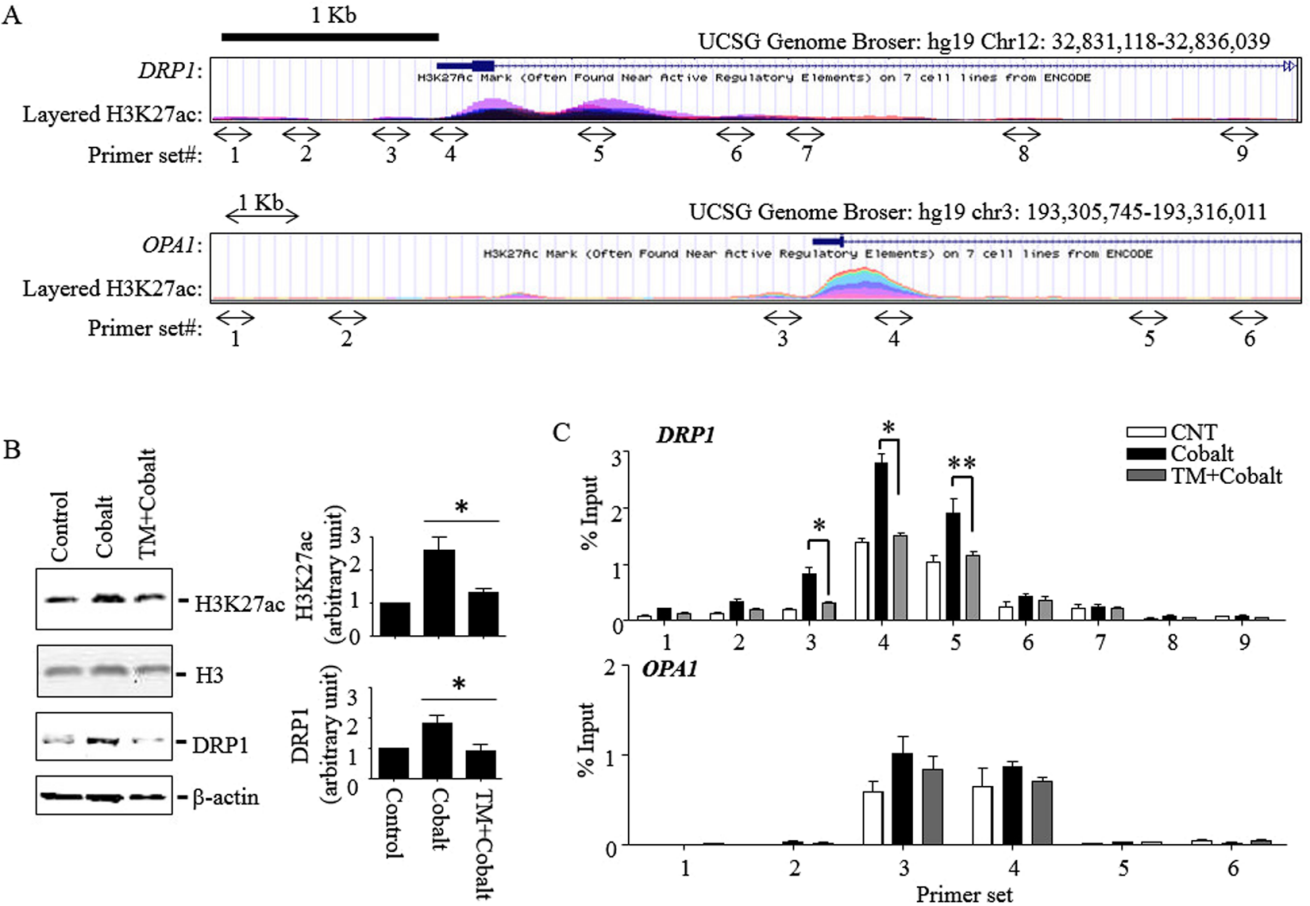
TM inhibits cobalt-induced DRP1 expression and decreases the level of H3K27ac association with the *DRP1* promoter. (**A**) Snapshot images of the ENCODE ChIP-sequence database showing the human *DRP1* and *OPA1* promoter regions and H3K27ac association. Arrows indicate locations of primer sets used for H3K27ac ChIP-qPCR analysis. (**B**,**C**) HK-2 cells were exposed to cobalt (300 µM) for 7 h and total cell lysates were extracted as described in “*Methods*”. (**B**) H3K27 acetylation levels and DRP1 expression were measured by Western blots using anti-H3K27ac and DRP1 antibodies (left panel). Immunoblots against H3 and β-actin were used as loading controls. H3K27ac and DRP1 band intensities were analyzed by NIH images J program (right panel). Data were shown as mean ± S.D. (n = 3; *p < 0.05; Student’s *t*-test). (**C**) H3K27ac association with *DRP1* and *OPA1* promoter regions was analyzed by CHIP-qPCR assay using anti-H3K27ac and ChIP primers targeting genomic regions shown in A. ChIP efficiency is represented as % of input DNA recovered by immunoprecipitation in two independent experiments (*P < 0.01; **P < 0.05; Student’s *t*-test).

Cobalt and hypoxia have been shown to regulate gene expression through stabilizing the hypoxia-inducible factor (HIF)-1α^27,28^. Therefore, we examined if HDAC8 affected HIF-1α protein levels in these cells using Western blots (Supplemental Fig. S3). Consistent with previous reports, cobalt and H/R increased protein levels of HIF-1α. However, TM had no significant effects on the protein levels, suggesting that HDAC8 does not regulate expression of DRP1 and OPA1 genes through HIF-1α. Since H3K27ac is associated with active promoters and enhancers, and HDAC8 deacetylates H3K27ac^29^, it is possible that HDAC8 regulates DRP1 and OPA1 expression through targeting H3K27ac. Therefore, we first examined H3K27ac ChIP-sequence data of 7 different cell types in the promoter regions of *DRP1* and *OPA1* available through the ENCODE database. As shown in Fig. 7A, both DRP1 and OPA1 have high levels of H3K27ac association in their promoter regions. First, we examined the levels of H3K27ac and DRP1 expression in HK-2 cells exposed to cobalt and cobalt plus TM. Cobalt induced both H3K27ac and DRP1 expression levels in HK-2 cells, which was inhibited by TM (Fig. 7B). Since HDAC8 is recruited to genomic regions in a gene-specific manner^30,31^, we further examined the level of H3K27ac association with the DRP1 and OPA1 promoter regions using ChIP-qPCR analysis (Fig. 7C). Indeed, the levels of H3K27ac association with the *DRP1* promoter region were increased by cobalt, which was prevented by TM. No significant changes were detected in the *OPA1* promoter region. These results suggest that HDAC8, at least in part, suppresses DRP1 expression through targeting H3K27ac associated with the *DRP1* promoter region.

## Discussion

Here, we showed that HDAC8 protected HK-2 cells from cytotoxicity induced by cobalt and H/R. TM, which is an N-acylthiourea derivative that selectively activates HDAC8 in high potency^32^, inhibited mitochondrial dysfunction (Fig. 2) and fission (Fig. 3A). Intact HDAC8 is constitutively active, but its catalytic activity within cells is expected to be suboptimal due to high intracellular K^+^ concentrations^33^. TM was shown to increase wild-type HDAC8 activity up to 12-fold at a 10 µM concentration^32^, likely through binding two distinct sites of HDAC8 that strengthens substrate binding and enhances catalytic activity^15^. To date, contradictory effects of TM on cytotoxicity have been reported, which could be due to different doses of TM and/or cell types used^15,34^. TM at 25 µM prevents cell cycle arrest and cell death induced by anthrax lethal toxin in human monocytic THP-1 cells^34^; whereas, TM at higher doses (40–80 µM) were shown to be toxic in neuroblastoma SH-YS5Y cells^15^. This study found that TM at 25–50 µM concentrations enhanced cell viability of HK-2 cells (Fig. 1A). Although statistically insignificant, a slight increase of cytotoxicity by TM at 50 µM was observed. We could not examine the effect of TM at higher than 50 µM concentrations since it started precipitating in the cell culture media. At this moment it is not clear whether the cytotoxic effect of TM was due to a dual effect HDAC8 or an off-target effect of TM.

HDAC8, although considered to be a class I HDAC, is distinct from other class I HDACs in multiple characteristics: localization in both cytoplasmic and nuclear compartments, high flexibility in substrate binding, post-translational modifications, and sensitivity to intracellular K^+^ concentrations^6,35^. HDAC8 is required for cell survival^36^ and its overexpression is associated with cancer cell proliferation^37^. HDAC8 is also involved in resistance to cell death and cell cycle arrest induced by anthrax lethal toxin, which cleaves and inactivates mitogen-activated protein kinase kinases through silencing mitochondrial cell death proteins^38^ and activating the cell survival Akt signaling cascade^39^. Also, knocking down HDAC8 by siRNA decreases cell proliferation in renal tubular cells^40^. In line with these studies, this study using HK-2 cells showed that overexpression of HDAC8 (Fig. 3B) or knocking down of HDAC8 (Fig. 1C) prevented or enhanced, respectively, mitochondrial dysfunction and cytotoxicity.

Mitochondrion is a dynamic organelle undergoing constant fission and fusion processes for structural and functional maintenance. However, pronounced mitochondrial fission triggered by cobalt, hypoxia, mitochondrial depolarization and other cellular stresses can lead to mitochondrial dysfunction and cell death^23,25,41^. Particularly, persistent mitochondrial fission is implicated in sustained tubular cell damage after ischemia-reperfusion acute kidney injury^42^. Key players involved in the mitochondrial fission process include, among others, DRP1, OPA1 and OMA1^25^. OPA1 is an inner mitochondrial membrane protein responsible for membrane fusion and *cristae* remodeling. Activity of OPA1 is regulated by proteolytic processing mediated by the zinc metalloproteases OMA1^43,44^. OMA1 resides in the inner membrane space of mitochondria as a dormant homo-oligomeric complex that can be activated by cellular stresses^45^. Active OMA1 cleaves OPA1 and leads to the cessation of inner membrane fusion. We found that TM significantly induced expression of OPA1 mRNA in HK-2 cells exposed to cobalt and H/R (Fig. 4), which, in part, contributed to the inhibitory effect of TM on mitochondrial fission. Previously, it was shown that the Akt-mTOR-NF-κB signaling cascade prevents mitochondrial fission and cell death through induction of OPA1 expression in neuronal and myocardial cells^46,47^. Since HDAC8 activates Akt in macrophages^34^, TM may have induced OPA1 mRNA expression through activating the Akt signaling cascade in HK-2 cells. Further studies are needed to address the involvement of the Akt-mTOR-NF-κB signaling cascade in OPA1 expression and cell survival mediated by HDAC8.

DRP1 is a cytosolic GTPase, which is recruited to the outer membrane of mitochondria through binding to multiple receptors, such as mitochondrial fission factor (MFF), FIS1 and mitochondrial elongation factor 1/2 (MIEF1/2), and forming a ring-like oligomerization at the focal point of mitochondrial fission^48^. Translocation of DRP1 to mitochondria is regulated by multiple routes, including transcriptional regulation^49,50^ and post-translational modifications of DRP1 by phosphorylation, SUMOylation, *O*-ClcNAcylation and S-nitrosylation^48^. Critical roles of DRP1 in mitochondrial fission, cytochrome C release and apoptotic cell death have been demonstrated using DRP1-deficient cells, siRNAs and the DRP1 inhibitor Mdivi-1 in kidney, brain and cardiac ischemia-reperfusion injury models^51–56^. DRP1 is also involved in necrotic programmed cell death, where the necroptosome, comprised of receptor-interacting protein kinase (RIPK)1/3 and mixed lineage kinase domain-like, activates phosphoglycerate mutase 5 (PGAM5), which in turn dephosphorylates and increases DRP1 GTPase activity^41^. In fact, necrotic programmed cell death is the main culprit of ischemic renal cell death^57^, whereby the DRP1 inhibitor Mdivi-1 prevents mitochondrial fission and cell death induced by renal ischemia-reperfusion^53^. We found that cobalt and H/R induced expression of DRP1, which was significantly prevented by TM (Fig. 4). Knocking down DRP1 by siRNA also prevented loss of mΔΨ and mitochondrial fission induced by cobalt and H/R (Fig. 5). Therefore, it is possible that HDAC8 prevents both apoptotic and necrotic programmed cell death induced by cobalt and H/R through downregulating DRP1 expression.

We further examined the mechanism of HDAC8 in the regulation of DRP1 expression. HDAC8 is highly expressed in renal proximal tubular cells, and knocking down HDAC8 by siRNA induces global histone H3 hyper-acetylation^40^, suggesting that HDAC8 deacetylates histone H3. More specifically, HDAC8 selectively deacetylates histone H3 lysine 27 residue (H3K27ac) in macrophages^29,38^. Here, we showed that H3K27ac levels were increased in HK-2 cells exposed to cobalt and H/R in a TM-sensitive manner (Fig. 7B), indicating that HDAC8 also targets H3K27ac in HK-2 cells. Since H3K27ac is a hallmark of active enhancers and promoters^58,59^, and HDAC8 is recruited to specific genomic sites^30^ in a cell type-dependent fashion^31^, we further examined if HDAC8 decreased H3K27ac levels at the promoters of *DRP1* and *OPA1*. As shown in Fig. 7C, cobalt induced the association of H3K27ac only at the promoter region of *DRP1*, but not to *OPA1*, in a TM-sensitive manner. These results suggest that HDAC8 inhibits DRP1 expression through selectively deacetylating H3K27ac at the *DRP1* promoter. HDAC8 has also been shown to regulate transcription through targeting various non-histones such as structural maintenance of chromosome 3, p53, protein phosphatase (PP) 1, heat shock proteins, α-actin, human ever-shorter telomeres 1B, estrogen-related receptor-α and possibly multiple other proteins^35,60,61^. Therefore, it is still possible that HDAC8 regulates transcription through mechanisms independent of histone deacetylation. Involvement of non-histones in the regulation of DRP1 and OPA1 expression by HDAC8 remains to be determined.

Despite intense research during the last decades, effective agents for preventing or treating tissue damage induced by ischemia are yet to be developed. Although most class I HDACs seem to play pathological roles^3,8^, this study showed that HDAC8, which is most distinct and insensitive to the class I-specific HDAC inhibitors, played a protective role in cobalt- and H/R-induced cytotoxicity. Therefore, we speculate that activating HDAC8 is beneficial for cell survival during ischemia-reperfusion of the kidney. However, due to the limitations of this study in using *in vitro* cell culture systems, the physiological role of HDAC8 in ischemia-reperfusion injury of the kidney needs to be confirmed in *in vivo* ischemia-reperfusion studies.

## Methods

### Reagents

The HDAC8 activator TM-2-51 (1-Benzoyl-3-phenyl-2-thiourea), the DRP1 inhibitor Mdivi-1 and cobalt chloride were purchased from Sigma-Aldrich (Oakville, Canada). The HDAC8-specific inhibitor PCI-34051 was purchased from Cayman Chemical (Burlington, Canada). Tetramethylrhodamine methyl ester perchlorate (TMRM) and Mito-Tracker^®^ Red CM-H2X ROS were obtained from Sigma-Aldrich and Invitrogen (Molecular Probes), respectively. Antibodies for mitochondrial voltage-dependent anion channel (VDAC) and p38 mitogen-activated protein kinases (p38) were purchased from Cell Signaling Technologies (Danvers, MA). Antibodies for H3K27ac, pan-histone H3 and HIF-1α were obtained from Active Motif (Carlsbad, CA), Bio Vision (Milpitas, CA) and Novus Biologicals (Littleton, CO), respectively. Antibodies for cytochrome C and DRP1 were obtained from Santa Cruz Biotechnology (Dallas, TX). The HDAC8-EGFP plasmid was prepared as previously described^38^. The HDAC8-H180R-EGFP plasmid was constructed after cloning the mutant HDAC8 from hHDAC8-6His-pET20b-H180R^62^, using the PCR primers: 5′-AGATTCTCGAGATGGAAGAACCGGAAGAACC-3′ and 5′-TTCAAGAATTCGAGAACCACGACCTTCGATAACA-3′ and inserting into the pEGFP-N1 vector using the XhoI and EcoR1 restriction enzymes.

### Cell culture

Human renal proximal tubular HK-2 cells (ATCC, Manassas, VA) were cultured in keratinocyte serum-free medium (K-SFM; GIBCO by Life Technologies) supplemented with bovine pituitary extract (0.05 mg/mL), epidermal growth factor (5 ng/mL) and 5% fetal bovine serum (Sigma-Aldrich). Rat renal proximal tubular NRK-52E cells (ATCC, Manassas, VA) were cultured in DMEM with 10% fetal bovine serum. Cells were grown at 37 °C in a humidified 5% CO_2_ and atmospheric O_2_ incubator.

### Cell viability assay

An MTT (3-(4,5-dimethylthiazol-2-yl)-2,5-diphenyltetrazolium bromide) assay was used to measure cytotoxicity, as described previously^63^. Briefly, HK-2 and NRK-52E cells were cultured in Dulbecco’s modified Eagle’s medium (DMEM) containing 2.5% FBS in 96-well plates for 16–18 h and subsequently treated with cobalt (CoCl_2_; Sigma-Aldrich) or dimethyl sulfoxide (DMSO: drug vehicle) in media for the time indicated. For H/R, cells were cultured with serum-free HK-2 cell culture media (for HK-2 cells) or serum-free DMEM (for NRK-52E cells) in a hypoxia chamber (HypOxygen, HypOxyststion H85; 0.2% O_2_, 5% CO_2_, balanced with N_2_ and humidified at 37 °C) for 24 h. These cells were then returned to a standard cell culture incubator (5% CO_2_ and atmospheric O_2_) at 37 °C for the time indicated. MTT was then added at a final concentration of 0.5 mg/ml. After incubating at 37 °C for an additional 3 h, culture media were carefully aspirated and 100 µl of DMSO was added to dissolve crystals. Optical density of each well was analyzed using a microplate reader (Synergy H4 Hybrid Reader; BioTek Instruments Inc.) at a 570 nm wavelength. The percentage of cytotoxicity was estimated by comparing the optical density of treated to non-treated cells (assumed 100% viability).

### Transfection of plasmids or small interfering (si)RNAs

Vector control (EGFP-N1), HDAC8-EGFP and HDAC8-H180R-EGFP plasmids, and human DRP1-specific (Invitrogen, Catalog No. 10620318–9, DNM1LHSS115288) and HDAC8-specific (Invitrogen, Catalog No. 10620318–9, HDAC8HSS125194) siRNAs were transfected into HK-2 cells using Lipofectamine 2000 or Lipofectamine RNAi Max kit (Invitrogen), according to the manufacturer’s instructions. Briefly, cells were plated on 6-well plates for 16–18 h and then transfected with 2 µg of plasmid DNA using Lipofectamine 2000. For siRNA transfection, siRNAs (120 pmole) were transfected into cells using Lipofectamine RNAi Max kit. After incubating with DNA or siRNA for 5 h, fresh media were replaced and cells further cultured for 24 h.

### Preparation of total cell lysates and subcellular fractionation

Total cell lysates were prepared as described previously^63^. Briefly, cell pellets were suspended in ice cold lysis buffer containing 20 mM MOPS, 2 mM EGTA, 5 mM EDTA, 1 mM Na_3_VO_4_, 40 mM β-glycerophosphate, 30 mM NaF, 20 mM sodium pyrophosphate, 0.1% SDS, 1% Triton X-100, pH7.2, and a protease inhibitor cocktail (Roche) for 10 min on ice. Lysates were then mixed with 4× SDS-PAGE sample buffer containing 5% (v/v) of β-mercaptoethanol, incubated in a block heater at 96 °C for 5 min, and loaded for SDS-PAGE. Subcellular cytosolic and mitochondrial fractions were purified using a modified sucrose gradient method, as described previously^63^. Briefly, cells were homogenized mechanically using a glass Dounce homogenizer in STE buffer (250 mM sucrose, 50 mM Tris, 1 mM EDTA, 1 mM NaF, 0.1 mM sodium orthovanadate, and protease inhibitor cocktail [pH 7.4]). The homogenates were centrifuged at 750 × g for 5 min to remove cell debris and nuclei, and supernatants were spun at 13,000 × g for 20 min to obtain mitochondrial pellets. Supernatants were then further spun at 20,000 × g for 30 min to obtain the cytoplasmic fraction. All steps were performed on ice or at 4 °C.

### Immunoblotting

Immunoblotting was performed as previously reported^63^. Briefly, cell lysates were separated by SDS-polyacrylamide gels and subsequently transferred onto nitrocellulose membranes (BioRad). After blocking with 5% (w/v) skim milk for 1 h at room temperature, the membranes were incubated overnight with primary antibodies and the next day washed three times with 1 × TTBS (20 mM Tris, 150 mM NaCl, pH 7.5) containing 0.07% Tween 20. The membranes were then incubated with secondary antibodies for 1 h at room temperature and images were developed using the enhanced chemiluminescence detection system (ECL; Thermo Scientific).

### Quantitative real-time PCR

Gene-specific mRNA amounts were estimated by quantitative real-time PCR (qPCR) as previously described^63^. Briefly, total cellular RNAs were isolated using TRIzol (Ambion by Life Technologies) and reverse transcribed by the Moloney murine leukemia virus (M-MuLV) reverse transcriptase kit (New England Biotechnology), according to the manufacturer’s instructions. To quantify mRNA expression, qPCR analyses were conducted using a Rotor-Gene RG3000 quantitative multiplex PCR instrument (Montreal Biotech) and PowerUp SYBR^TM^ Green Master Mix (Applied Biosystems/ThermoFisher Scientific). The data were normalized by expression of the GAPDH housekeeping gene. Primers used for qPCR are listed in Supplemental Table 1.

### Mitochondrial transmembrane potential (ΔΨm) measurement

TMRM was used to measure ΔΨm. Cells exposed to cobalt or H/R were washed with pre-warmed fresh media and stained with TMRM (200 nM) for 30 min at 37 °C according to the ImmunoChemistry Technologies, MitoPT^®^ TMRM assay protocol. Cells were then examined by FACS (Beckman Coulter) and the data acquired was analyzed using CytExpert software and FlowJo 7.6.5.

MitoTracker staining and mitochondrial fragmentation. Mito-Tracker Red CM-H_2_X ROS (Invitrogen, Molecular Probes) was used to stain mitochondria and observe morphology, as previously reported^63^. Stained cells were observed under a fluorescence microscope (Olympus) at 600x magnification. Images were then acquired using QCapture Pro software and the number of cells showing mitochondrial fragmentation was counted. The proportion of cells with fragmented mitochondria was calculated as a percentage of the total number of cells counted.

### Chromatin immunoprecipitation (ChIP) analysis

ChIP analysis was conducted as described previously^38^ using a H3K27ac antibody (Active Motif). Purified DNAs were subjected to quantitative real-time PCR analysis using Power SYBR Green PCR Master Mix (Applied Biosystems) and primers targeting the promoter/intragenic region of the human DRP1 and OPA1 genes. Primers used for ChIP analysis are listed in Supplemental Table 1. Data are presented as percent of enrichment with the precipitated target sequence compared with input DNA.

### Statistical analysis

Statistical analysis was carried out using GraphPad Prism 4.0 (GraphPad Software). Student’s *t*-tests or one-way ANOVA with Tukey multiple comparison tests were performed as stated in the figure legends. Statistical significance was defined as p < 0.05.

## Electronic supplementary material

Supplemental Table and Figures
