# Supplementary material for: Histone deacetylase 8 protects human proximal tubular epithelial cells from hypoxia-mimetic cobalt- and hypoxia/reoxygenation-induced mitochondrial fission and cytotoxicity
Source: Sci Rep. 2018 Jul 27;8:11332. doi: 10.1038/s41598-018-29463-x (PMC6063935; doi:10.1038/s41598-018-29463-x)
Supplement: Supplementary file 1 — Supplemental Table and Figures [file 41598_2018_29463_MOESM1_ESM.pdf]

Histone deacetylase 8 protects human proximal tubular epithelial cells from hypoxia-mimetic cobalt- and hypoxia/reoxygenation-induced mitochondrial fission and cytotoxicity.

Soon-Duck Ha, Ori Solomon, Masoud Akbari, Alp Sener and Sung Ouk Kim

Supplementary Table 1. Primers used for qPCR and ChIP assays.

Gene	Primer ID	Primer sequence (5’>3’)
GAPDH	qPCR	F: 5’-ACCCACTCCTCCACCTTTG-3’<br>R: 5’-CTCTTGTGCTCTTGCTGGG-3’
DRP1	qPCR	F: 5`-GAGTTACTGGTGAAGCGGCA-3`<br>R: 5`-ATTGCCACTAAGTTATGGACCA-3’
FIS1	qPCR	F: 5`-AAGAGCACGCAGTTTGAGTAC-3`<br>R: 5`-CTGGGGCTCTGTCTGCAGC-3`
OMA1	qPCR	F: 5`-GGCACTTTTGTGCCAGTGGA-3`<br>R: 5’-GGCCATGCAGGCTATCAACG-3`
OPA1	qPCR	F: 5`-GCTGAACGCAGTATTGTTACAG-3`<br>R: 5`-TATAGCTTCAATGCTTTCAGAGCT-3’
HDAC8	qPCR	F: 5`-ATTCTCTACGTGGATTTGGATC-3`<br>R: 5`-ATGCCATCCTGAATGGGCACA-3`
DRP1<br>(ChIP)	#1	F: 5`-AGCAAAGAGTGAGTAAGGCAGTT-3`<br>R: 5`-AGAGTATTGGAGTTTTACAGGGGA-3`
	#2	F: 5`-GAACCCGCGTATTCTAACCT-3`<br>R: 5`-TTCTCTAGAAGGACGGGTTGG -3`
	#3	F: 5`-AGACACACCATTGCGCCTT-3`<br>R: 5`-GCAGGCTCTAGTCAAAGGCTA-3`
	#4	F: 5`-CCCCATTCATTGCCGTGG-3`<br>R: 5`-CTACGACGATTTGAGGCAGC-3`
	#5	F: 5`-GCTTTCGTGAGACGGGTGTT-3`<br>R: 5`-CGTTCTCGAAACCTGGTGGAA-3`
	#6	F: 5`-TTAGTGGCTATCCAAACACTGGA-3`<br>R: 5`-TTTCCCAATCCTATCGCAGACC-3`
	#7	F: 5`-TGCTCACTCTAACCACAATGTTT-3`<br>R: 5`-TGGAAAGTTTCTAGTGTGCAGC-3`
	#8	F: 5`-CCATTACTTTGTGATGTCTGCCA-3`<br>R: 5`-ACTGCCGATAGGTATACTAGGC-3`
	#9	F: 5`-GGTGAGGGACCATAGGTGATTC-3`<br>R: 5`-TGGTACTGGATGGTAGTATGCC-3`
OPA1<br>(ChIP)	#1	F: 5`-TTTATGGTGTTACCTTCCGTGA-3`<br>R: 5`-AACTGGGACATAGCCCTAAATG-3`
	#2	F: 5`-TAATGTGACCCAAAGCTTCACCT-3`<br>R: 5`-ATTGGTGCTGAAGTGTGAATGG-3`
	#3	F: 5`-GCCTTGACGAGGACCATTTC-3`<br>R: 5`-CAGTTAGGCCAGTAACGGGT-3`
	#4	F: 5`-TCTTTCCCTAGCCCGCTGAT-3`<br>R: 5`-CGTTCACGACCTTGCATCAAA-3`
	#5	F: 5`-CTAGCACTGAGCATTGCAACTTT-3`<br>R: 5`-GATACCATCAAAATGCTAAGGCG-3`
	#6	F: 5`-GTAAAGCTAGAGACTGCCCAGG-3`<br>R: 5`-TTACGGAGGGCACCATAGGC-3`

Histone deacetylase 8 protects human proximal tubular epithelial cells from hypoxia-mimetic cobalt- and hypoxia/reoxygenation-induced mitochondrial fission and cytotoxicity.

Soon-Duck Ha, Ori Solomon, Masoud Akbari, Alp Sener and Sung Ouk Kim

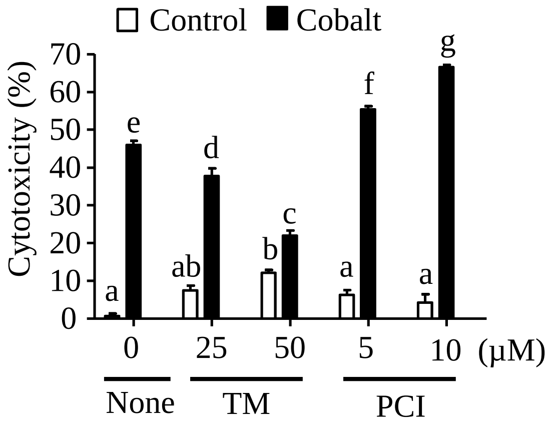

Supplemental Fig. S1. Rat renal proximal tubular NRK-52E cells were treated with 150 µM of cobalt in the presence or absence of the HADAC8 activator TM-2-51 (TM; 25 & 50 µM), inhibitor PCI34051 (PCI; 5 & 10 µM) or drug vehicle DMSO for 20-24 h. Cytotoxicity was measured by MTT assay as described in "*Methods*". Data are expressed as mean ±S.D. [n=3; $p < 0.05$ by one-way ANOVA with Tukey multiple comparison test; columns accompanied by the same letter are not significantly different from each other].

Histone deacetylase 8 protects human proximal tubular epithelial cells from hypoxia-mimetic cobalt- and hypoxia/reoxygenation-induced mitochondrial fission and cytotoxicity.

Soon-Duck Ha, Ori Solomon, Masoud Akbari, Alp Sener and Sung Ouk Kim

A

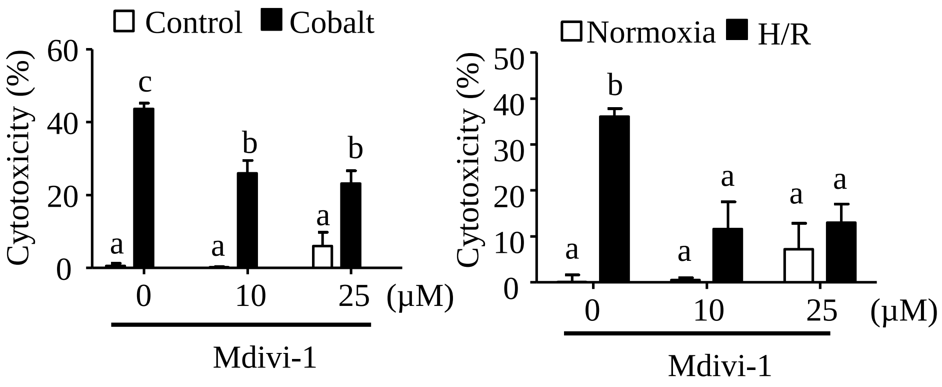

B

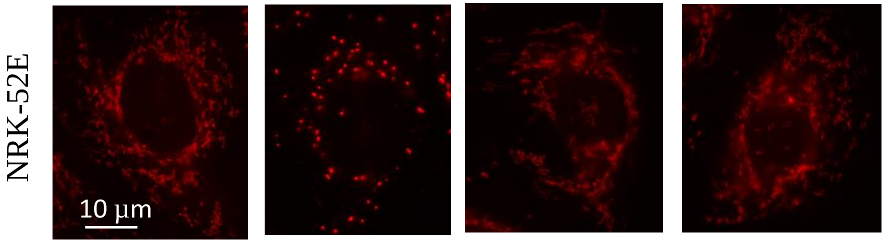

Supplemental Fig. S2. A. Rat renal proximal tubular NRK-52E cells were treated with 150 µM of cobalt in the presence or absence of Mdivi-1 (10 & 25 µM) or drug vehicle DMSO for 20-24 h. Cytotoxicity was measured by MTT assay as in Figures 1 and 5. Data are expressed as mean ± S.D. [n=3; $p < 0.05$ by one-way ANOVA with Tukey multiple comparison test; columns accompanied by same letter (a, b or c) are not significantly different from each other]. **B**. Similarly, HK-2 cells were treated with cobalt (300 µM) in the absence or presence of Mdivi-1 (10 & 25 µM) for 16 h and stained with MitoTracker®-Red as in Figure 3. Cells were then visualized (600x magnification). One representative image of three independent experiments is shown.

Histone deacetylase 8 protects human proximal tubular epithelial cells from hypoxia-mimetic cobalt- and hypoxia/reoxygenation-induced mitochondrial fission and cytotoxicity.

Soon-Duck Ha, Ori Solomon, Masoud Akbari, Alp Sener and Sung Ouk Kim

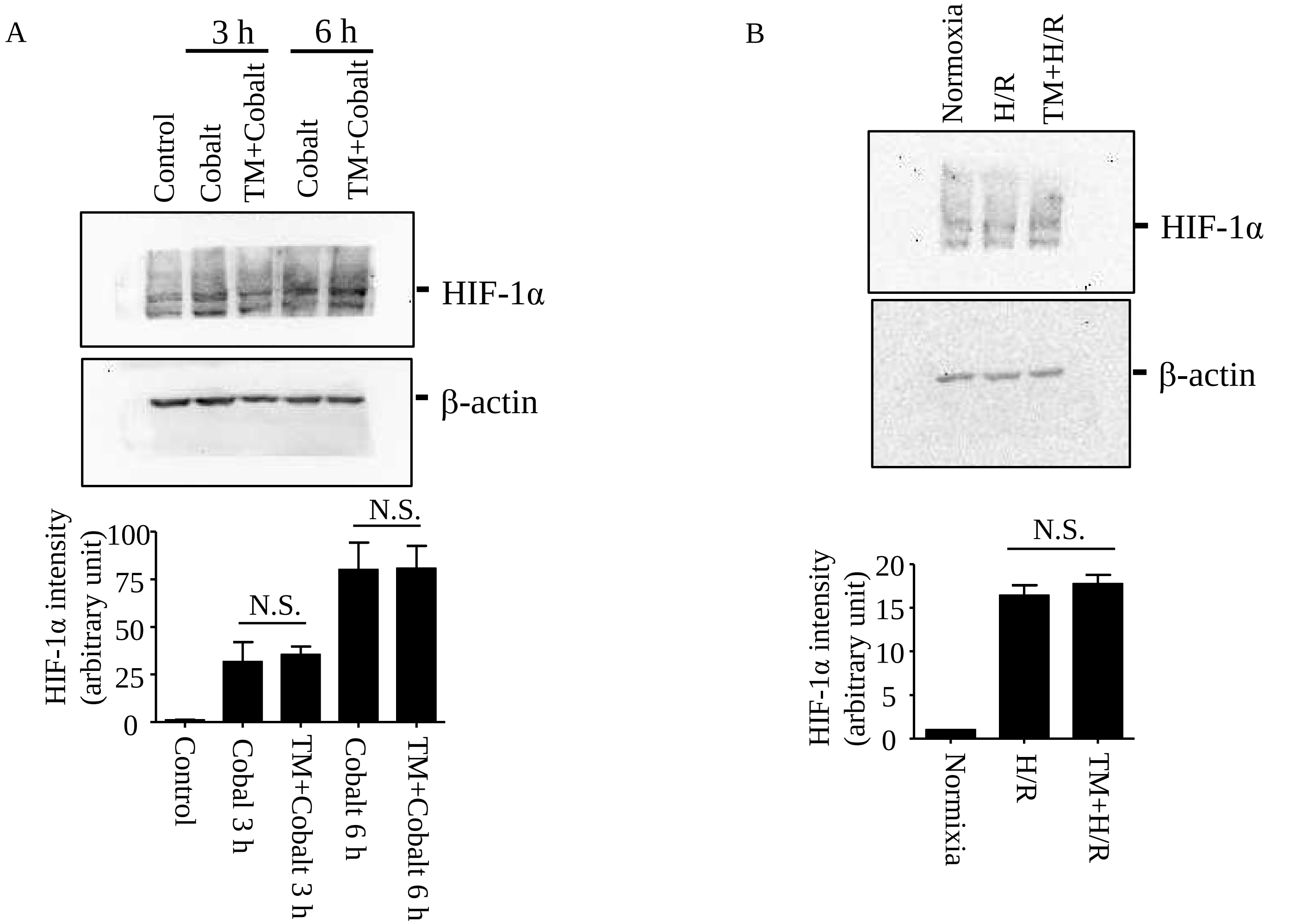

Supplemental Fig. S3. **A-B**. HK-2 cells were exposed to cobalt (300 μM) for the time indicated (A), or incubated in the hypoxia chamber (0.2% O_2, 5% CO_2 & 95% N_2 at 37 °C) for 24 h, followed by incubation in a standard incubator (~21% O2, 5% CO2 & ~74% N2 at 37 °C) for the next 3 h (B), in the absence or presence of TM (25 μM). HIF-1α protein levels were measured by Western blots. Immunoblot against β-actin was used as the loading control. HIF-1α band intensity was analyzed by NIH images J program (A-B, lower panel). N.S., not significant by Student's *t*-test.

Histone deacetylase 8 protects human proximal tubular epithelial cells from hypoxia-mimetic cobalt- and hypoxia/reoxygenation-induced mitochondrial fission and cytotoxicity.

Soon-Duck Ha, Ori Solomon, Masoud Akbari, Alp Sener and Sung Ouk Kim

Supplemental Fig. S4. Full-length images of Western blots

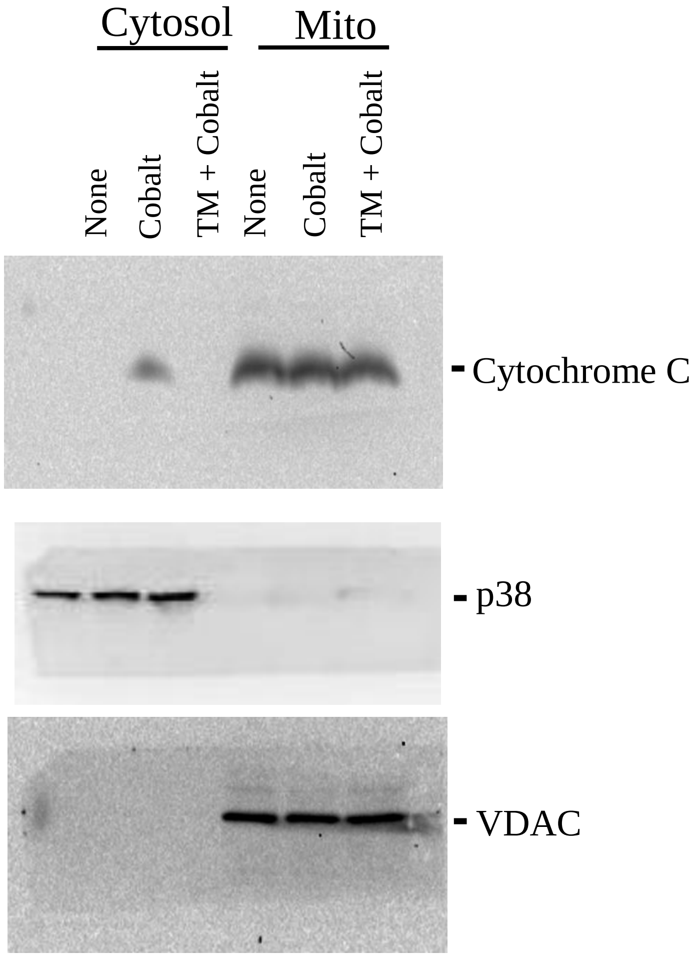

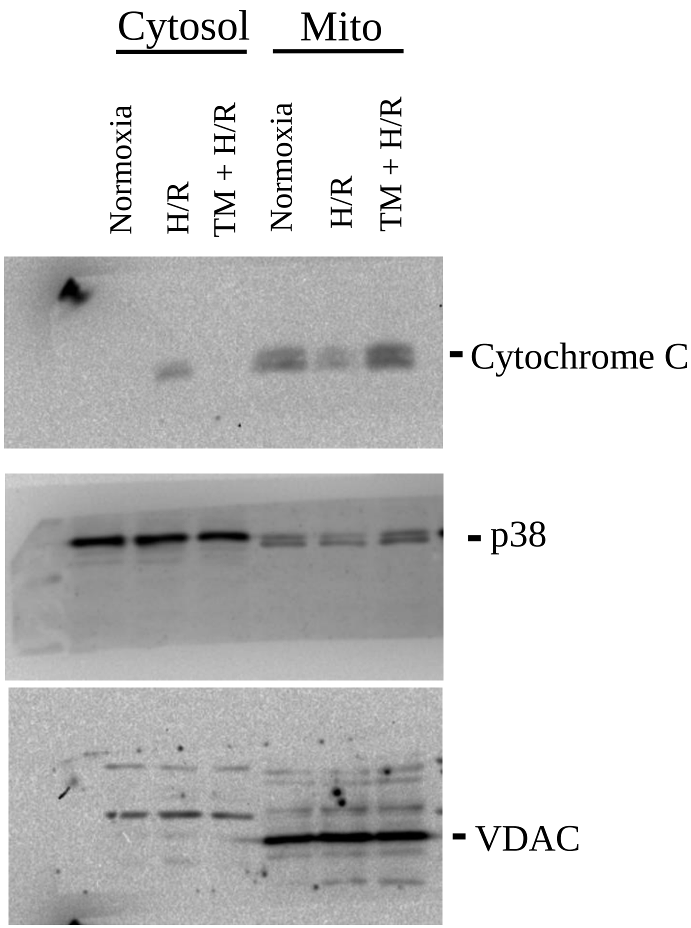

Full-length images of Western blots shown in Fig. 2B.

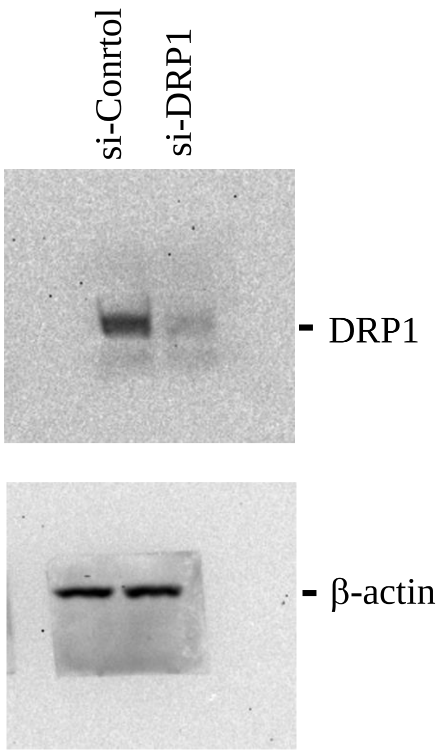

Full-length images of Western blots shown in Fig. 6A.

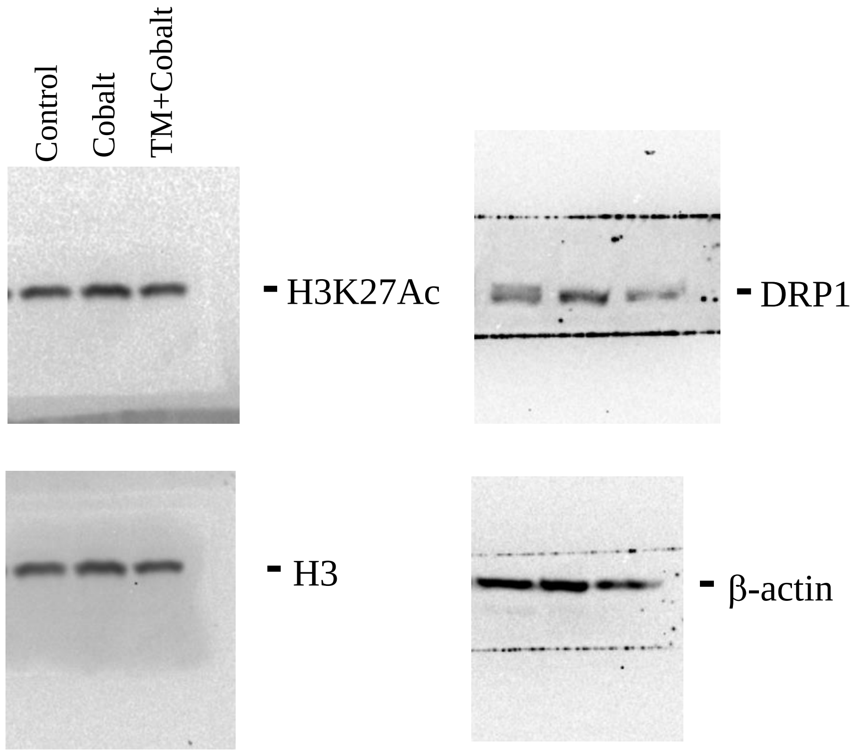

Full-length images of Western blots shown in Fig. 7B.
